# Increasing Polarity in Tacrine and Huprine Derivatives: Potent Anticholinesterase Agents for the Treatment of Myasthenia Gravis

**DOI:** 10.3390/molecules23030634

**Published:** 2018-03-11

**Authors:** Carles Galdeano, Nicolas Coquelle, Monika Cieslikiewicz-Bouet, Manuela Bartolini, Belén Pérez, M. Victòria Clos, Israel Silman, Ludovic Jean, Jacques-Philippe Colletier, Pierre-Yves Renard, Diego Muñoz-Torrero

**Affiliations:** 1 Laboratory of Pharmaceutical Chemistry (CSIC Associated Unit), Faculty of Pharmacy and Food Sciences, and Institute of Biomedicine (IBUB), University of Barcelona, Av. Joan XXIII 27-31, E-08028 Barcelona, Spain; cgaldeano@ub.edu; 2 Institut de Biologie Structurale, Université Grenoble Alpes, Centre National de la Recherche Scientifique (CNRS)-Commissariat à l’Énergie Atomique (CEA) (UMR 5075), F-38054 Grenoble, France; coquelleni@ill.fr; 3 Large-Scale Structures Group, Institut Laue-Langevin, 71 Avenue des Martyrs, 38000 Grenoble, France; 4 Laboratory COBRA (UMR 6014), Normandie Université, UNIROUEN, Institut National des Sciences Appliquées (INSA) Rouen, CNRS, 76000 Rouen, France; monika.bouet@gmail.com; 5 Department of Pharmacy and Biotechnology, Alma Mater Studiorum University of Bologna, Via Belmeloro 6, I-40126 Bologna, Italy; manuela.bartolini3@unibo.it; 6 Department of Pharmacology, Therapeutics and Toxicology, Neuroscience Institute, Autonomous University of Barcelona, E-08193 Barcelona, Spain; belen.perez@uab.cat (B.P.); victoria.clos@uab.cat (M.V.C.); 7 Department of Neurobiology, Weizmann Institute of Science, 76100 Rehovot, Israel; israel.silman@weizmann.ac.il

**Keywords:** acetylcholinesterase inhibitors, butyrylcholinesterase inhibitors, quinolinium compounds, triazoles, structural biology, copper-catalyzed azide-alkyne cycloaddition, click chemistry

## Abstract

Symptomatic treatment of myasthenia gravis is based on the use of peripherally-acting acetylcholinesterase (AChE) inhibitors that, in some cases, must be discontinued due to the occurrence of a number of side-effects. Thus, new AChE inhibitors are being developed and investigated for their potential use against this disease. Here, we have explored two alternative approaches to get access to peripherally-acting AChE inhibitors as new agents against myasthenia gravis, by structural modification of the brain permeable anti-Alzheimer AChE inhibitors tacrine, 6-chlorotacrine, and huprine Y. Both quaternization upon methylation of the quinoline nitrogen atom, and tethering of a triazole ring, with, in some cases, the additional incorporation of a polyphenol-like moiety, result in more polar compounds with higher inhibitory activity against human AChE (up to 190-fold) and butyrylcholinesterase (up to 40-fold) than pyridostigmine, the standard drug for symptomatic treatment of myasthenia gravis. The novel compounds are furthermore devoid of brain permeability, thereby emerging as interesting leads against myasthenia gravis.

## 1. Introduction

Myasthenia gravis is an autoimmune disease that is associated with antibodies directed against nicotinic acetylcholine receptors (AChRs) on the postsynaptic membrane at the neuromuscular junction, or against other proteins, such as muscle-specific tyrosine kinase (MuSK), low-density lipoprotein receptor-related protein 4 (LRP4), and agrin, which are involved in AChR clustering on the postsynaptic membrane and in structural maintenance of the neuromuscular synapse [1,2]. Antibodies against AChRs may block these receptors by binding to the acetylcholine-binding site, may reduce the number of available receptors in the membrane by internalization, and may activate the complement cascade, leading to destruction of the postsynaptic muscle membrane and, hence, negatively affecting AChR function [2,3]. Failure of neuromuscular transmission leads to fluctuating skeletal muscle fatigue and weakness upon repeated contraction, which is the main clinical hallmark of myasthenia gravis. Weakness affects various muscle groups, such as extraocular muscles, which control eye movements, bulbar muscles in the mouth and throat, responsible for speech and swallowing, and limb and axial muscles [1].

The worldwide prevalence of myasthenia gravis has been estimated to be 40–180 per million people, with 10% being children and adolescents, and an annual incidence of 4–12 per million people, with little geographical variation [1,2,4]. Even though myasthenia gravis remains a rare disease, its incidence and prevalence are increasing, particularly in the elderly [5], likely as a result of improved recognition and diagnosis, due to the widespread availability of autoantibody tests, and of increased life expectancy [4].

**Figure 1:**
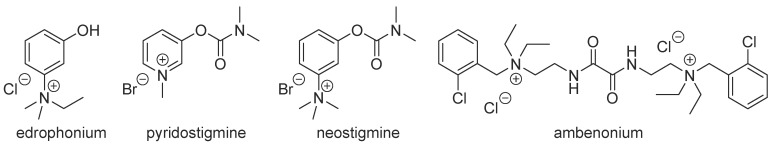
Chemical structures of peripherally-acting AChEIs used for diagnosis and symptomatic treatment of myasthenia gravis.

Diagnostic testing and symptomatic treatment of myasthenia gravis are based on restoration of cholinergic transmission at the neuromuscular junction by means of peripherally-acting inhibitors of acetylcholinesterase (AChE), the enzyme responsible for the hydrolysis of the neurotransmitter acetylcholine at the synapse [6]. Inhibition of acetylcholine breakdown renders the neurotransmitter more available for nicotinic AChR stimulation, which alleviates muscle weakness by improving neuromuscular transmission [7]. The short-acting AChE inhibitor (AChEI) edrophonium chloride (Enlon^®^, Tensilon^®^, Figure 1) is used for diagnosis of myasthenia gravis, because it leads to dramatic amelioration of the functioning of a particularly weak muscle group immediately after administration. Other AChEIs, such as pyridostigmine bromide (Mestinon^®^), neostigmine bromide (Prostigmin^®^), and ambenonium dichloride (Mytelase^®^) (Figure 1), which were introduced in the early 1950s, are used for the symptomatic treatment of early and mild myasthenia gravis [3,7,8]. Patients who do not appropriately respond to symptomatic treatment should be treated with immunosuppressive drugs, which target the underlying pathological immune response, with azathioprine being one of those most widely used [1,2].

Commercially available AChEIs often permit patients suffering from myasthenia gravis to function normally, without the need for immunosuppressive drugs. However, their use is accompanied by a number of muscarinic receptor-mediated side-effects, including abdominal cramps, diarrhea, increased gastric and bronchial secretions, salivation, lacrimation, nasal discharge, sweating, increased urination, and bradycardia, which can result in discontinuation of the treatment [7]. Thus, novel AChE inhibitors with more favorable efficacy/safety profiles are being developed for the symptomatic treatment of myasthenia gravis.

To ensure a peripheral effect and prevent blood‒brain barrier (BBB) crossing and, hence, potential central cholinergic side-effects, the design of novel AChEIs against myasthenia gravis is commonly based on the introduction of permanently charged quaternary nitrogens [9]. A number of alkylammonium [10], piperidinium [11,12], pyridinium [13], quinolinium [14], and isoquinolinium [15] derivatives have been developed as potential drug candidates against myasthenia gravis or as reversal agents of neuromuscular blockade in surgical anesthesia. These compounds usually exhibit AChE inhibitory potencies in the low micromolar to low nanomolar range. It is noteworthy that some of these compounds are formally derived, by quaternization, from centrally-acting AChE inhibitors that are being used for treatment of Alzheimer’s disease, such as donepezil [11,12]. Permanently charged derivatives of the anti-Alzheimer drug galantamine have been also reported as potent peripherally-acting AChEIs [16].

**Scheme 1:**
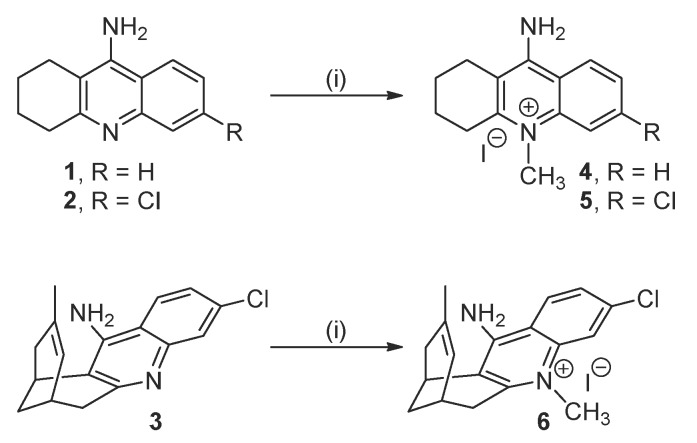
Synthesis of tacrine-, 6-chlorotacrine-, and huprine-based quinolinium derivatives **4**–**6**. Reagents and conditions: (i) MeI, methyl ethyl ketone, reflux, overnight, 91% yield (**4**), 91% yield (**5**), 77% yield (**6**).

Tacrine (**1**, Scheme 1) was the first AChEI to be approved for treatment of Alzheimer’s disease [17]. Its chlorosubstituted derivatives 6-chlorotacrine, **2** [18], and huprine Y, **3** [19,20,21], are very potent AChEIs that were also developed as candidate drugs against Alzheimer’s disease.

**Scheme 2:**
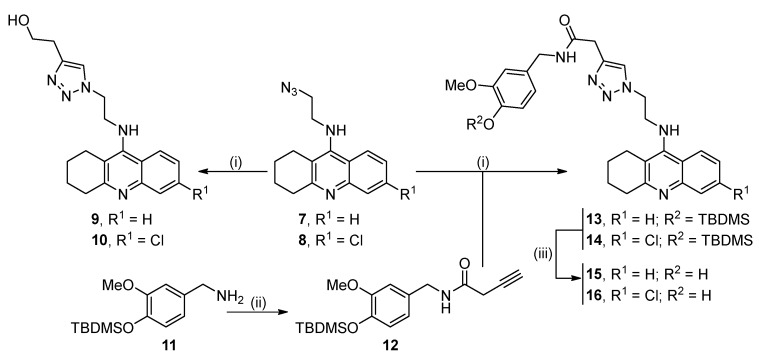
Synthesis of tacrine-based triazole derivatives **9**, **10**, **15**, and **16**. Reagents and conditions: (i) alkyne (3-butyn-1-ol or **12**), CuI, acetonitrile, r.t., 16 h, 63% yield (**9**), 81% yield (**10**), 99% yield (**13**), 60% yield (**14**); (ii) 3-butynoic acid, EDC·HCl, CH_2_Cl_2_, r.t., 2 h, 36% yield; (iii) CSA, MeOH, r.t., 18 h (for **15**, 15% yield) or TBAF, THF, 0 °C, 1 h, then r.t., 1 h (for **16**, 69% yield).

In this study, we explored two different approaches to accessing more polar derivatives of tacrine, 6-chlorotacrine, and huprine Y, which may serve as new peripherally-acting AChEIs of potential value for treatment of myasthenia gravis. First, we envisaged the classic approach, involving design of permanently charged quinolinium salts (compounds **4**–**6**, Scheme 1). As an alternative approach, we also explored the introduction of non-charged polar moieties attached to a tacrine or 6-chlorotacrine core. To this end, we considered the introduction of a 1,2,3-triazole ring (compounds **9** and **10**, Scheme 2). The 1,2,3-triazole ring can be readily installed through the prototypic click reaction, the Huisgen 1,3-dipolar cycloaddition reaction of alkynes and azides [22]. It is a very stable moiety, non-hydrolytically cleavable, essentially non-oxidizable and non-reducible, with a very large dipole moment, and with two out of its three nitrogen atoms able to act as weak hydrogen bond acceptors, so that they can be involved in interactions with biological targets [23]. Indeed, previous studies have highlighted additional favorable interactions of the 1,2,3-triazole group with residues in the AChE active-site gorge, when attached to a tacrine moiety by a two-carbon linker. These additional interactions seem to account for the efficacy of the in situ click chemistry approach (KTGS, kinetic target-guided synthesis) for the synthesis of dual binding-site AChE inhibitors [24,25,26,27]. In addition, in some target compounds we decided to introduce a second polar moiety, namely a polyphenol-like moiety at the end of a triazole-containing tether chain (compounds **15** and **16**, Scheme 2), with the double purpose of increasing polarity and producing dual site binding to AChE, i.e., simultaneous interaction with both the catalytic anionic site (CAS), at the bottom of the active-site gorge, and with the peripheral anionic site (PAS), at the mouth of the gorge [28], which should result in increased inhibitory potency.

Here we report the synthesis of the above-mentioned novel polar derivatives of tacrine, 6-chlorotacrine, and huprine Y, and the in vitro evaluation of their inhibitory activities against human AChE (hAChE) and human butyrylcholinesterase (hBChE), a second human cholinesterase that hydrolyzes acetylcholine in the blood and in the central nervous system. To assess the peripheral distribution and discard potential central cholinergic effects of these lead compounds, their brain permeability was also assessed in vitro. Furthermore, the three-dimensional structures of complexes of several of these compounds with *Torpedo californica* AChE (*Tc*AChE) were also solved, in order to shed light on their mechanism of action.

## 2. Results and Discussion

### 2.1. Synthesis of the Target Compounds

The new quinolinium iodide derivatives **4**–**6** were prepared in good yield by reaction of tacrine, 6-chlorotacrine, and huprine Y, respectively, with excess iodomethane in refluxing methyl ethyl ketone [29] (Scheme 1).

The synthesis of the triazole-based compounds **9**, **10**, **15**, and **16** started from the known azidoalkyltacrines **7** [30] and **8** [31], which, in turn, were readily prepared by reaction of 9-chloro-1,2,3,4-tetrahydroacridine or 6,9-dichloro-1,2,3,4-tetrahydroacridine with 2-aminoethanol, followed by conversion of the resulting hydroxyethyl tacrine derivatives to the corresponding mesylates [32], and a final nucleophilic substitution reaction with sodium azide in acetonitrile at 80 °C. Copper-catalyzed azide-alkyne cycloaddition reaction of azides **7** and **8** with 3-butyn-1-ol afforded the target triazole-containing tacrines **9** and **10** in 63% and 81% yield, respectively (Scheme 2).

For the synthesis of the target triazole-based compounds **15** and **16**, the new alkyne **12** was first prepared by amide coupling of the know amine **11** [33] with 3-butynoic acid (Scheme 2). Copper(I)-catalyzed Huisgen reaction of alkyne **12** with azides **7** and **8** afforded in good yields the *O*-TBDMS-protected triazole intermediates **13** and **14**, which were finally converted into the target triazole-containing tacrines **15** and **16** upon deprotection with camphorsulfonic acid (CSA) in MeOH or with tetrabutylammonium fluoride (TBAF) in THF, respectively (Scheme 2).

### 2.2. Biological Profiling of the Target Compounds

The inhibitory activities of the novel compounds against human recombinant AChE and human serum BChE were evaluated using the method by Ellman et al. [34], using as reference compounds pyridostigime, the most widely used AChE inhibitor for treatment of myasthenia gravis, and tacrine, 6-chlorotacrine, and huprine Y, the apolar brain-permeable parent compounds.

Interestingly, all the novel compounds exhibited hAChE and hBChE inhibitory potencies in the low micromolar to submicromolar, or even low nanomolar range, with most being more potent than or equipotent to pyridostigmine (Table 1).

On the basis of the results obtained, some structure‒activity relationships (SAR) can be derived: (i) quaternization of the potent inhibitors tacrine, 6-chlorotacrine, and huprine Y to the quinolinium derivatives **4**–**6** results in a drop in their inhibitory potency towards both hAChE and hBChE; (ii) the ranking of potencies for the quinolinium derivatives remains the same as that found for the noncharged parent compounds, i.e., huprine derivative **6** > 6-chlorotacrine derivative **5** > tacrine derivative **4** for hAChE inhibition and tacrine derivative **4** > huprine derivative **6** > 6-chlorotacrine derivative **5**, for hBChE inhibition, with the well-known contribution of the chlorine atom on the benzene ring of tacrine and huprine derivatives, which is positive for hAChE inhibition [35], and detrimental for hBChE inhibition [36,37], being retained in the quinolinium derivatives; (iii) introduction of the triazole-containing side chain at position 9 of tacrine and 6-chlorotacrine results in a slight drop in inhibitory activity towards both hAChE and hBChE for compounds **9** and **10**; (iv) however, when the triazole-containing side chain is terminated with a polyphenol-like aromatic ring, the resulting compounds, **15** and **16**, are slightly more potent hAChE and hBChE inhibitors than the parent tacrine and 6-chlorotacrine, likely as a result of multi-site binding within both these cholinesterases; (v) the positive and negative effects of the chlorine atom in the tacrine derivatives on hAChE and hBChE inhibitory activities, respectively, also operates in the triazole series. 

Inhibition of AChE at the neuromuscular junction results in an extended lifetime of the neurotransmitter ACh in the synaptic cleft, which repetitively activates the nicotinic AChRs that, in myasthenia gravis, are affected by autoantibodies. This compensates for the reduction in nicotinic AChR density, thereby rescuing muscle function. Conversely, inhibition of BChE, which is localized on the surface of terminal Schwann cells, key components of the neuromuscular junction, not only does not have an effect on synaptic ACh lifetime, but depresses ACh release into the synaptic cleft, via activation of α7 nicotinic AChRs, which co-localize with BChE on terminal Schwann cells [38,39]. Thus, selective inhibitors of AChE are the preferred option for the functional improvement of muscle function in myasthenia gravis, as compared to non-selective inhibitors, for which concomitant inhibition of BChE counteracts the positive effect produced by AChE inhibition. In this context, the huprine and triazole derivatives **6**, **10** and **16** are those with the most potent and selective hAChE inhibitory activity, with IC_50_ values of 59.2 nM, 200 nM and 7.18 nM, respectively, and selectivities for hAChE over hBChE inhibition of 55, 16, and 40, respectively. Most important, these compounds are 23-, 7- and 192-fold more potent and 37-, 11- and 27-fold more selective hAChE inhibitors than pyridostigmine, the most widely used drug for treatment of myasthenia gravis and other myasthenic syndromes [40]. 

To assess whether the two strategies to increase polarity that were used in the design of the target compounds could produce the expected selective peripheral distribution, preventing entry into the central nervous system, we evaluated the brain permeability of these compounds by the widely used in vitro parallel artificial membrane permeability assay for BBB (PAMPA-BBB) [41], using a lipid extract of porcine brain membrane. As expected, the experimentally determined permeability values (*Pe*) of the new polar tacrine and huprine derivatives were, in most cases, in the desired range of low BBB penetration. The sole exception was the huprine-based quinolinium derivative **6**, which, despite showing a clearly lower *Pe* value than the parent noncharged compound, huprine Y (*Pe* 10.90 vs. 23.8 (10^−6^ cm/s)), was nevertheless still in the range of high BBB permeation (Table 1, see also Table 2 for *Pe* values of commercial drugs uses in assay validation).

Even though most of the novel compounds would be expected to display the desired low brain penetration, more thorough physicochemical and pharmacokinetic characterization will be necessary to assess their druglikeness and to address potential ADMET issues, especially in the case of the higher molecular weight compounds **9**, **10**, **15**, and **16** [42].

### 2.3. Structural Characterization of Complexes of Compounds ***5***, ***6***, and ***16*** with TcAChE

To obtain insight into the binding mode of the novel quinolinium and triazole derivatives within the active-site gorge of AChE, these compounds were subjected to co-crystallization trials with *Tc*AChE and structure determination was attempted for the crystalline complexes thus obtained. Crystals were soaked for 12 h in mother liquor supplemented with a 1 mM solution of the compound. Only the co-crystals obtained with compounds **5**, **6** and **16** diffracted satisfactorily, thus permitting solution of their crystal structures. Data collection, structure determination and refinement are further detailed under Materials and Methods (see also Table 3).

**Figure 2:**
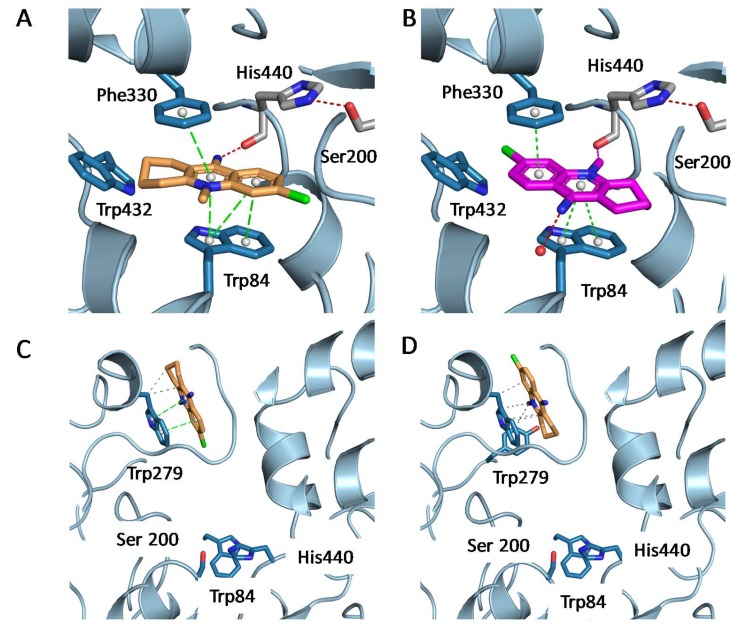
Binding mode of **5** within the *Tc*AChE active site (**A**,**B**), and at the PAS (**C**,**D**). In the active site, the major pose is represented in (**A**), with the conjugated system of **5** π-stacked between Phe330 and Trp84. However, the Cl atom does not point towards Trp432, as usually observed for 6-chlorotacrine derivatives, and the nitrogen of the exocyclic amino group is H-bonded to the carbonyl group of catalytic His440. A minor pose has also been modeled, with the Cl atom towards the hydrophobic pocket. In this orientation, the methyl group makes close contact (2.7 Å) with catalytic His440, and potentially interacts *via* a weak H-bond [45]. At the peripheral site, two orientations are also observed, and were refined with identical occupancies (0.5). Hydrophobic interactions are represented as dashed gray lines, π-stacking interactions are in green, and hydrogen bonds are in red.

In the crystal structure of *Tc*AChE complexed with the 6-chlorotacrine-based quinolinium derivative **5**, we found three molecules of **5** bound to each of the two monomers present in the asymmetric unit. In the active site, the binding mode of **5** is different from that of 6-chlorotacrine in a hybrid derivative described by Nepovimova et al. (pdb code 4TVK) [43]. While the conjugated system of the molecule is sandwiched between Trp84 and Phe330, making a π-stacking interaction with Trp84, the orientation of the chlorine atom is quite unusual. Indeed, several crystal structures of acetylcholinesterase with 6-chlorotacrine, or even with the closely related compound, huprine X, the 9-ethyl analog of huprine Y [35], all share a common feature, in which the chlorine atom fits into a hydrophobic groove contributed by Phe330, Tyr334, Trp432, Met437 and Ile439 (see also, below, the complex of *Tc*AChE with the 6-chlorotacrine-based triazole derivative **16**). In the *Tc*AChE/**5** complex, the chlorine atom faces the opposite side of the gorge, pointing towards the main-chain of residues 116–118 (Figure 2A). To fully validate this new orientation, a Polder map [44], the new generation of the Phenix omit map, was calculated. This map validated our modeling of **5** within the active site of *Tc*AChE, with a 13.2 σ electron density peak coinciding with the position of the modeled chlorine. In this orientation, the nitrogen of the exocyclic amino group H-bonds to the carbonyl of catalytic His440 (2.73 Å), and the methyl group points towards the entrance of the gorge. The loss of the important interactions of the chlorine atom in the above-mentioned hydrophobic pocket is most probably at the origin of the 300-fold drop of potency when compared to 6-chlorotacrine.

A closer inspection of the Polder map revealed a bump in the density that was not satisfactorily modeled with this unique orientation. In the refinement cycles following the addition of **5** in the model, this positive density bump was retained in the mFo-DFc maps. This bump would perfectly fit the position of the Cl atom in the usual orientation of 6-chlorotacrine within the AChE active site. We decided, therefore, to model a second, minor, orientation in the active site of *Tc*AChE (Figure 2B). In this orientation, the nitrogen atom of the exocyclic amino group is H-bonded to water molecules, while the methyl group faces the main chain of catalytic Ser200 (2.74 Å). This close contact of the methyl group with the protein is probably the reason that this conformation is poorly populated.

Other large electron density blobs in the mFo-DFc maps indicated the presence of a second copy of **5** at the PAS. **5** was modeled, making a π–π stacking with Trp279, but in two alternate conformations, with the Cl atom pointing either into or out of the gorge (Figure 2C,D). Finally, a third molecule of **5** was modeled sandwiched between the aforementioned **5** molecules at the PAS and a symmetry-related protein molecule of the crystal (Figure 2C). The presence of this third copy of **5** at the PAS thus appears to be a consequence of crystal packing interactions (not shown).

**Figure 3:**
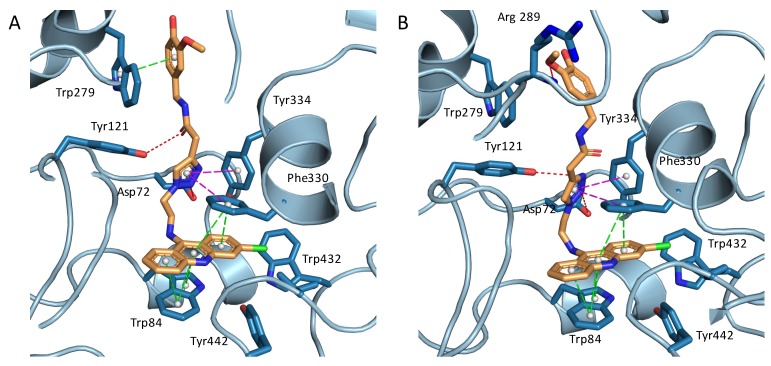
Binding mode of **16** within the active site of *Tc*AChE in the two copies of the monomer in the asymmetric unit. The binding of the 6-chlorotacrine moiety and of the linker up to the triazole ring are similar. The binding of the phenolic moiety differs. In (**A**), the phenolic moiety is stabilized via a parallel *π*-stacking interaction, and is thus more stable than in monomer B (**B**), in which only the ether oxygen atom of the phenolic moiety is H-bonded to the main chain nitrogen atom of Arg289. Parallel and perpendicular *π*-stacking interactions are shown in green and magenta respectively, while H-bonds are depicted in red.

In the crystal structure of *Tc*AChE complexed with the 6-chlorotacrine-based triazole derivative **16**, the 6-chlorotacrine moiety binds as previously observed, with the conjugated system π-stacked to Trp84, and the Cl atom fitting into the hydrophobic groove comprising Phe330, Tyr334, Trp432, Met437, and Ile439. Along the linker, the triazole ring is engaged in two perpendicular π-stacking interactions with Tyr334 and Phe300, and in H-bonds with Tyr121 and Asp72. From this point onwards, the observed conformations of the phenolic moiety of **16** differ in the two TcAChE monomers of the asymmetric unit. In monomer A (Figure 3A), the phenolic moiety makes a π-stacking interaction with Trp279 in the PAS. In monomer B (Figure 3B), the ether oxygen of the phenolic moiety H-bonds to the main carbonyl of Arg289.

**Figure 4:**
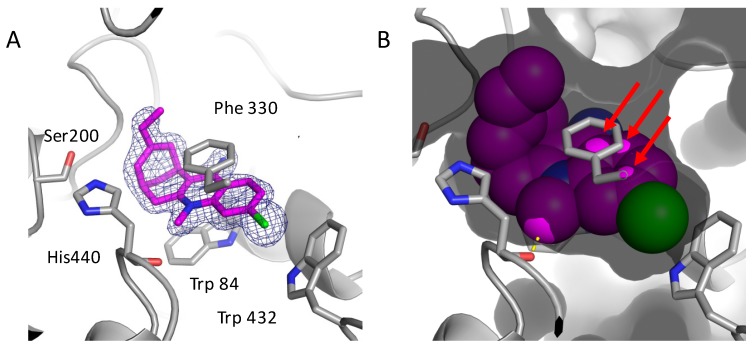
Binding of **6** to *Tc*AChE. (**A**) 2mFO-DFc map, contoured at 1 σ, of **6** within the active site of *Tc*AChE. Clear electron density is observed for the extra methyl group of **6**; (**B**) **6** is depicted in space-filling format, and the gorge surface is shown in gray. A close contact is observed with the carbonyl group of His440 (yellow dashed line). Introduction of the methyl group also resulted in reorientation of some atoms of **6** that are in close proximity to Phe330 (red arrows in panel (**B**)).

Finally, the crystal structure of the complex of *Tc*AChE with the huprine-based quinolinium derivative **6** shows that the modified huprine moiety binds at the bottom of the gorge, in the choline-binding pocket, via π-stacking interactions with both Trp84 and Phe330 (Figure 4), similarly to the parent huprines W, X and Y [35,46,47]. However, to accommodate the additional methyl group, the huprine plane is slightly shifted away from the catalytic His440, and upwards towards Phe330. When compared to the previously solved structure of the *Tc*AChE/huprine X complex (pdb code 1e66) [35], the chlorine atom barely moves (0.3 Å, for an overall RMSD of 0.2 Å for 222 superimposed Ca atoms), but the deviation increases up to 0.7 Å at the other extremity of the huprine moiety. The extra methyl group constrains accommodation of the huprine moiety in the active site, resulting in close contact with the carbonyl group of His440 (2.7–2.8 Å) and Phe330 (distances of 3.2 and 3.3 Å observed in the π-stacking interactions, while the short-range repulsion distance between two carbon atoms is 3.4 Å). Taken together, these repulsive close contacts, generated by the addition of the methyl group, could account for the 55-fold reduction in inhibitory potency of **6** relative to the parent huprine Y.

## 3. Materials and Methods

### 3.1. Chemistry 

#### General Methods

Solvents to be used for syntheses were purified with a dry solvent station MB-SPS-800 (MBraun) immediately prior to use. All reagents were obtained from commercial suppliers (Sigma Aldrich (Madrid, Spain; Saint Quentin Fallavier, France), Acros (Belgium), TCI (Japan)) unless otherwise stated, and used without further purification. The reactions were monitored by thin-layer chromatography (TLC) using silica gel (60 F254) plates. Compounds were visualized by UV irradiation and/or spraying with 1% aqueous KMnO_4_, followed by charring at 150 °C. Flash column chromatography was performed on silica gel 60 (230–400 mesh, 0.040–0.063 mm) and silica gel 200 (0.060–0.200 mm). Melting points of the new compounds were measured with a MFB 595010M Gallenkamp apparatus. ^1^H-NMR (300 or 400 MHz) and ^13^C-NMR (75 or 101 MHz) spectra were recorded on Bruker 300 (Billerica, Massachusetts, MA, USA) or Varian Mercury 400 spectrometers (Palo Alto, California, CA, USA) at the Centres Científics i Tecnològics of the University of Barcelona (CCiTUB) and at the Laboratory COBRA of the University of Rouen; chemical shifts are reported in ppm (δ scale) relative to residual solvent signals (CD_3_OD, DMSO-*d*_6_ or CDCl_3_ at 3.31/49.0, 2.50/39.5, or 7.26/77.1 ppm, respectively, in the ^1^H/^13^C-NMRspectra) and coupling constants are reported in Hertz (Hz). The *syn* (*anti*) notation of the protons at position 13 of the huprine derivative **6** means that the corresponding proton at position 13 is on the same (different) side of the quinoline moiety with respect to the cyclohexene ring. Mass spectra were recorded using the electrospray ionisation technique (ESI). High-resolution mass spectra (HRMS) were obtained with Varian MAT 311 or LC/MSD TOF Agilent Technologies spectrometers, using electrospray analysis. The purity of the novel compounds was determined by analytical HPLC on an Agilent 1100 Series instrument (method A, (NH_4_)_2_HPO_4_/MeOH pH 7.7, flow rate 1 mL/min, det. 254 nm) or on a Thermo Scientific Surveyor Plus instrument equipped with a PDA detector (method B, MeCN/H_2_O/0.10% TFA, flow rate 1 mL/min, det. 254 and 330 nm). Semi-preparative RP-HPLC was performed on a Thermo Scientific SPECTRASYSTEM liquid chromatography system (P4000) equipped with a UV-visible 2000 detector (method C, Varian Kromasil C18 column, 10 μm, 21.2 mm × 250 mm, with MeCN and 0.1% aq. TFA as eluents, 0% MeCN (5 min), followed by linear gradients of 0–30% (40 min), 30–40% (50 min), and 40–80% (110 min) MeCN, at a flow rate of 20.0 mL/min. Elution profiles were monitored at 250 and 340 nm.

*9-Amino-1,2,3,4-tetrahydro-10-methylacridin-10-ium iodide* (**4**). A solution of tacrine, **1** (300 mg, 1.51 mmol), in methyl ethyl ketone (25 mL) was heated to 80 °C, and then treated dropwise with iodomethane (6.67 mL, 15.2 g, 107 mmol). The reaction mixture was stirred under reflux overnight and cooled to room temperature. The resulting precipitate was collected by filtration under vacuum and was washed with methyl ethyl ketone (3 × 25 mL). The tacrine-based pyridinium derivative **4** (465 mg, 91% yield) was thus obtained as a white solid: m.p. > 290 °C (dec); IR (ATR) *ν* 3319, 3185 (N-H st), 1645, 1619, 1592, 1554, 1532 (ar-C-C, ar-C-N st); ^1^H-NMR (400 MHz, DMSO-*d*_6_) *δ* 1.75–1.90 (complex signal, 4H, 2-H_2_, 3-H_2_), 2.58 (m, 2H, 1-H_2_), 3.09 (m, 2H, 4-H_2_), 3.99 (s, 3H, 10-CH_3_), 7.69 (m, 1H, 7-H), 7.97 (m, 1H, 6-H), 8.16 (d, *J* = 8.8 Hz, 1H, 5-H), 8.42 (br s, 2H, 9-NH_2_), 8.54 (d, *J* = 8.0 Hz, 1H, 8-H); ^13^C-NMR (101 MHz, DMSO-*d*_6_) *δ* 20.3 (CH_2_), 21.5 (CH_2_), 23.9 (CH_2_) (C1, C2, C3), 29.0 (CH_2_, C4), 35.9 (CH_3_, 10-CH_3_), 110.8 (C, C9a), 115.6 (C, C8a), 118.2 (CH, C5), 123.9 (CH), 125.7 (CH) (C7, C8), 133.6 (CH, C6), 138.8 (C, C10a), 154.1 (C), 154.8 (C) (C4a, C9); HRMS (ESI), calculated for C_14_H_17_N_2_ [M]^+^ 213.1386, found 213.1339; HPLC purity 99.5% (*t*_R_ = 15.0 min, method A).

*9-Amino-6-chloro-1,2,3,4-tetrahydro-10-methylacridin-10-ium iodide* (**5**). It was prepared as described for **4**. From 6-chlorotacrine, **2** (300 mg, 1.29 mmol), the 6-chlorotacrine-based pyridinium derivative **5** (442 mg, 91% yield) was obtained as a white solid: m.p. >290 °C (dec); IR (ATR) *ν* 3301, 3187 (N-H st), 1642, 1615, 1588, 1553, 1520 (ar-C-C, ar-C-N st); ^1^H-NMR (400 MHz, DMSO-*d*_6_) *δ* 1.75–1.90 (complex signal, 4H, 2-H_2_, 3-H_2_), 2.55 (t, *J* = 6.0 Hz, 2H, 1-H_2_), 3.07 (t, *J* = 6.0 Hz, 2H, 4-H_2_), 3.97 (s, 3H, 10-CH_3_), 7.78 (dd, *J* = 9.2 Hz, *J′* = 2.0 Hz, 1H, 7-H), 8.15 (br s, 1H, 9-NH_A_), 8.28 (d, *J* = 2.0 Hz, 1H, 5-H), 8.54 (d, *J* = 9.2 Hz, 1H, 8-H), 8.95 (br s, 1H, 9-NH_B_); ^13^C-NMR (101 MHz, DMSO-*d*_6_) *δ* 20.2 (CH_2_), 21.4 (CH_2_), 23.9 (CH_2_) (C1, C2, C3), 29.0 (CH_2_, C4), 36.3 (CH_3_, 10-CH_3_), 111.5 (C, C9a), 114.2 (C, C8a), 117.9 (CH, C5), 126.0 (CH), 126.2 (CH) (C7, C8), 138.7 (C), 139.6 (C) (C6, C10a), 154.6 (C), 154.9 (C) (C4a, C9); HRMS (ESI), calculated for C_14_H_16_^35^ClN_2_ [M]^+^ 247.0997, found 247.0999; HPLC purity 99.7% (*t*_R_ = 17.7 min, method A).

*12-Amino-3-chloro-6,7,10,11-tetrahydro-5,9-dimethyl-7,11-methanocycloocta[b]quinolin-5-ium iodide* (**6**). It was prepared as described for **4**. From huprine Y, **3** (300 mg, 1.05 mmol), the huprine-based pyridinium derivative **6** (346 mg, 77% yield) was obtained as a white solid: m.p. 283–285 °C (dec); IR (ATR) *ν* 3314, 3188 (N-H st), 1649, 1611, 1585, 1522 (ar-C-C, ar-C-N st); ^1^H-NMR (400 MHz, DMSO-*d*_6_) *δ* 1.52 (s, 3H, 9-CH_3_), 1.77 (br d, *J* = 12.0 Hz, 1H, 13-H*_syn_*), 1.87 (d, *J* = 17.6 Hz, 1H, 10-H*_endo_*), superimposed in part 1.86–1.92 (m, 1H, 13-H*_anti_*), 2.40 (dd, *J* = 17.6 Hz, *J′* = 3.6 Hz, 1H, 10-H*_exo_*), 2.80 (br s, 1H, 7-H), 3.03 (d, *J* = 18.0 Hz, 1H, 6-H*_endo_*), 3.23 (dd, *J* = 18.0 Hz, *J′* = 6.0 Hz, 1H, 6-H*_exo_*), 3.40 (br s, 1H, 11-H), 3.97 (s, 3H, 5-CH_3_), 5.50 (br d, *J* = 5.6 Hz, 1H, 8-H), 7.80 (dd, *J* = 9.2 Hz, *J′* = 2.0 Hz, 1H, 2-H), 8.27 (d, *J* = 2.0 Hz, 1H, 4-H), 8.36 (br s, 1H, 12-NH_A_), 8.58 (d, *J* = 9.2 Hz, 1H, 1-H), 8.94 (br s, 1H, 12-NH_B_); ^13^C-NMR (101 MHz, DMSO-*d*_6_) *δ* 23.0 (CH_3_, 9-CH_3_), 26.0 (CH), 26.6 (CH) (C7, C11), 26.7 (CH_2_, C13), 34.7 (CH_2_), 35.7 (CH_2_) (C6, C10), 36.4 (CH_3_, 5-CH_3_), 114.6 (C), 115.1 (C) (C11a, C12a), 117.8 (CH, C4), 124.2 (CH, C8), 126.1 (CH), 126.2 (CH) (C1, C2), 132.8 (C, C9), 138.6 (C, C3), 139.7 (C, C4a), 153.7 (C), 154.1 (C) (C5a, C12); HRMS (ESI), calculated for C_18_H_20_^35^ClN_2_ [M]^+^ 299.1310, found 299.1316; HPLC purity 99.7% (*t*_R_ = 22.1 min, method A).

*2-{1-[2-(1,2,3,4-Tetrahydroacridin-9-ylamino)ethyl]-1H-1,2,3-triazol-4-yl}ethanol* (**9**). To a solution of azide **7** (50 mg, 187 µmol) in acetonitrile (HPLC grade, conc = 0.06 M), 3-butyn-1-ol (16 mg, 224 µmol) was added via a syringe. The reaction flask was then protected from light, and CuI (36 mg, 187 µmol) was introduced. The reaction mixture was stirred at room temperature for 16 h. The solvent was evaporated, and the crude product was directly purified by column chromatography (silica gel, CH_2_Cl_2_/MeOH mixtures, gradient elution, 100:0 to 95:5, 90:10 and 85:15) to afford the triazole derivative **9** (40 mg, 63% yield) as an orange-brown oil; *R*_f_ (CH_2_Cl_2_/MeOH 9:1) 0.53; ^1^H-NMR (300 MHz, CD_3_OD) *δ* 1.89–1.91 (m, 4H, tacrine 2-H_2_, 3-H_2_), 2.62 (t, *J* = 6.0 Hz, 2H, tacrine 1-H_2_), 2.77 (t, *J* = 6.9 Hz, 2H, 2-H_2_), 2.96 (t, *J* = 5.7 Hz, 2H, tacrine 4-H_2_), 3.60 (t, *J* = 6.9 Hz, 2H, 1-H_2_), 4.15 (t, *J* = 5.4 Hz, 2H, NH-C*H*_2_-CH_2_-N), 4.60 (t, *J* = 5.4 Hz, 2H, NH-CH_2_-C*H*_2_-N), 7.41 (ddd, *J* = 7.8 Hz, *J′* = 6.9 Hz, *J″* = 1.2 Hz, 1H, tacrine 7-H), 7.58 (s, 1H, triazole 5-H), 7.62 (ddd, *J* = 8.1 Hz, *J′* = 6.9 Hz, *J″* = 1.2 Hz, 1H, tacrine 6-H), 7.75 (d, *J* = 7.8 Hz, 1H, tacrine 8-H), 7.98 (d, *J* = 8.1 Hz, 1H, tacrine 5-H); ^13^C-NMR (75 MHz, CD_3_OD) *δ* 22.9 (CH_2_, tacrine C2), 23.6 (CH_2_, tacrine C3), 25.7 (CH_2_, tacrine C1), 29.7 (CH_2_, C2), 32.6 (CH_2_, tacrine C4), 48.8 (CH_2_, NH-*C*H_2_-CH_2_-N), 51.7 (CH_2_, NH-CH_2_-*C*H_2_-N), 61.9 (CH_2_, C1), 116.9 (C, tacrine C9a), 120.1 (C, tacrine C8a), 124.4 (CH, triazole C5), 124.5 (CH, tacrine C5), 125.3 (CH, tacrine C8), 125.8 (CH, tacrine C7), 131.3 (CH, tacrine C6), 144.7 (C, triazole C4), 146.2 (C, tacrine 10a), 154.2 (C, tacrine C9), 157.1 (C, tacrine C4a); MS (ESI+) 338 [M + H]^+^; HRMS (ESI+), calculated for C_19_H_24_N_5_O [M + H]^+^ 338.1981, found 338.1978; HPLC purity 99.9% (*t*_R_ = 17.4 min, method B).

*2-{1-[2-(6-Chloro-1,2,3,4-tetrahydroacridin-9-ylamino)ethyl]-1H-1,2,3-triazol-4-yl}ethanol* (**10**). It was prepared as described for **9**. From azide **8** (40 mg, 133 µmol), the triazole derivative **10** (40 mg, 81% yield) was obtained as an orange-brown oil; *R*_f_ (CH_2_Cl_2_/MeOH 9:1) 0.27; ^1^H-NMR (300 MHz, CD_3_OD) *δ* 1.89–1.91 (m, 4H, tacrine 2-H_2_, 3-H_2_), 2.61 (t, *J* = 6.0 Hz, 2H, tacrine 1-H_2_), 2.74 (m, 2H, 2-H_2_), 2.95 (t, *J* = 5.7 Hz, 2H, tacrine 4-H_2_), 3.65 (m, 2H, 1-H_2_), 4.11 (t, *J* = 5.7 Hz, 2H, NH-C*H*_2_-CH_2_-N), 4.59 (t, *J* = 5.7 Hz, 2H, NH-CH_2_-C*H*_2_-N), 7.36 (dd, *J* = 9.3 Hz, *J′* = 2.1 Hz, 1H, tacrine 7-H), 7.57 (s, 1H, triazole 5-H), 7.75 (d, *J* = 2.1 Hz, 1H, tacrine 5-H), 7.94 (d, *J* = 9.3 Hz, 1H, tacrine 8-H); ^13^C-NMR (75 MHz, CD_3_OD) *δ* 23.0 (CH_2_, tacrine C2), 23.6 (CH_2_, tacrine C3), 25.8 (CH_2_, tacrine C1), 29.7 (CH_2_, C2), 33.3 (CH_2_, tacrine C4), 48.8 (CH_2_, NH-*C*H_2_-CH_2_-N), 51.7 (CH_2_, NH-CH_2_-*C*H_2_-N), 61.9 (CH_2_, C1), 117.7 (C, tacrine C9a), 118.9 (C, triazole C4), 120.7 (C, tacrine C8a), 124.3 (CH, triazole C5), 125.1 (CH, tacrine C5), 125.9 (CH, tacrine C7), 126.4 (CH, tacrine C8), 136.6 (C, tacrine C6), 146.5 (C, tacrine 10a), 153.4 (C, tacrine C9), 159.2 (C, tacrine C4a); MS (ESI+) 372 [M + H]^+^; HRMS (ESI+), calculated for C_19_H_23_^35^ClN_5_O [M + H]^+^ 372.1591, found 372.1586; HPLC purity 99.1% (*t*_R_ = 17.9 min, method B).

*N-[4-(tert-Butyldimethylsilyloxy)-3-methoxybenzyl]-3-butynamide* (**12**). To a solution of [4-(*tert*-butyldimethylsilyloxy)-3-methoxyphenyl]methanamine, **11** (2.10 g, 7.85 mmol), in dichloromethane (40 mL), 3-butynoic acid (0.66 g, 7.85 mmol) and *N*-(3-dimethylaminopropyl)-*N′*-ethylcarbodiimide hydrochloride, EDC·HCl (1.18 g, 6.16 mmol), were successively added. The reaction mixture was stirred at room temperature for 2 h, diluted with water, and extracted with dichloromethane (2 × 100 mL). The combined organic phases were washed with water, then with brine, dried over anhydrous MgSO_4_, and concentrated under reduced pressure. The crude product was purified by flash column chromatography (silica gel, cyclohexane/EtOAc, 80:20 to 70:30) to yield the alkynamide **12** (950 mg, 36% yield) as a yellow-orange solid. The isomerization allene derivative was also isolated (430 mg, 16% yield) as an off-white solid. **12**: ^1^H-NMR (300 MHz, CDCl_3_) *δ* 0.14 [s, 6H, OSi(C*H*_3_)_2_C(CH_3_)_3_], 0.98 [s, 9H, OSi(CH_3_)_2_C(C*H*_3_)_3_], 2.33 (t, *J* = 2.7 Hz, 1H, 4-H), 3.26 (d, *J* = 2.7 Hz, 2H, 2-H_2_), 3.79 (s, 3H, 3′-OCH_3_), 4.39 (d, *J* = 5.7 Hz, 2H, CONHC*H*_2_), 6.67–6.85 (complex signal, 3H, 2′-H, 5′-H, 6′-H); ^13^C-NMR (75 MHz, CDCl_3_) *δ* −4.5 [2CH_3_, OSi(*C*H_3_)_2_C(CH_3_)_3_], 18.6 [C, OSi(CH_3_)_2_*C*(CH_3_)_3_], 25.8 [3CH_3_, OSi(CH_3_)_2_C(*C*H_3_)_3_], 27.5 (CH_2_, C2), 43.9 (CH_2_, CONH*C*H_2_), 55.6 (CH_3_, 3′-OCH_3_), 74.4 (CH, C4), 77.5 (C, C3), 111.9 (CH, C2′), 120.2 (CH, C5′), 121.0 (CH, C6′), 131.1 (C, C1′), 144.7 (C, C4′), 151.2 (C, C3′), 166.0 (C, C1); MS (ESI+) 334 [M + H]^+^.

*N-[4-(tert-Butyldimethylsilyloxy)-3-methoxybenzyl]-2-{1-[2-(1,2,3,4-tetrahydroacridin-9-ylamino)ethyl]-1H-1,2,3-triazol-4-yl}acetamide* (**13**). It was prepared as described for **9**. From azide **7** (40 mg, 150 µmol), the triazole derivative **13** (89 mg, 99% yield) was obtained as a brown oil; *R*_f_ (CH_2_Cl_2_/MeOH 9:1) 0.30; ^1^H-NMR (300 MHz, CD_3_OD) *δ* 0.05 [s, 6H, OSi(C*H*_3_)_2_C(CH_3_)_3_], 0.90 [s, 9H, OSi(CH_3_)_2_C(C*H*_3_)_3_], 1.65–1.95 (complex signal, 4H, tacrine 2-H_2_, 3-H_2_), 2.59 (t, *J* = 6.0 Hz, 2H, tacrine 1-H_2_), 2.93 (t, *J* = 6.0 Hz, 2H, tacrine 4-H_2_), 3.57 (s, 2H, 2-H_2_), 3.72 (NH), 3.77 (s, 3H, Ph-3-OCH_3_), 4.06 (t, *J* = 5.4 Hz, 2H, NH-C*H*_2_-CH_2_-N), 4.24 (s, 2H, CONHC*H*_2_), 4.60 (t, *J* = 5.4 Hz, 2H, NH-CH_2_-C*H*_2_-N), 6.64–6.75 (complex signal, 2H, Ph-5-H, Ph-6-H), 6.82 (s, 1H, Ph-2-H), 7.37 (br t, *J* = 7.5 Hz, 1H, tacrine 7-H), 7.58 (br t, *J* = 7.5 Hz, 1H, tacrine 6-H), 7.69 (s, 1H, triazole 5-H), 7.74 (br d, *J* = 8.4 Hz, 1H, tacrine 8-H), 7.90 (br d, *J* = 8.5 Hz, 1H, tacrine 5-H); ^13^C-NMR (75 MHz, CD_3_OD) *δ* −3.6 [2CH_3_, OSi(*C*H_3_)_2_C(CH_3_)_3_], 18.8 [C, OSi(CH_3_)_2_*C*(CH_3_)_3_], 23.1 (CH_2_, tacrine C2), 23.7 (CH_2_, tacrine C3), 25.8 (CH_2_, tacrine C1), 26.2 [3CH_3_, OSi(CH_3_)_2_C(*C*H_3_)_3_], 33.1 (CH_2_, tacrine C4), 33.6 (CH_2_, C2), 44.2 (CH_2_, CONH*C*H_2_), 48.8 (CH_2_, NH-*C*H_2_-CH_2_-N), 51.7 (CH_2_, NH-CH_2_-*C*H_2_-N), 56.3 (CH_3_, Ph-3-OCH_3_), 112.5 (CH, Ph-C2), 116.1 (CH, Ph-C5), 117.6 (C, tacrine C9a), 120.7 (Ph-C6, tacrine C8a), 124.2 (CH, tacrine C5), 125.4 (CH, triazole C5), 125.6 (CH, tacrine C7), 126.4 (CH, tacrine C8), 130.7 (CH, tacrine C6), 131.1 (C, Ph-C1), 142.9 (C, triazole C4), 145.9 (C, Ph-C4), 146.8 (C, tacrine C10a), 148.9 (C, Ph-C3), 153.2 (C, tacrine C9), 158.1 (C, tacrine C4a), 171.4 (C, C1); MS (ESI+) 601 [M + H]^+^.

*N-[4-(tert-Butyldimethylsilyloxy)-3-methoxybenzyl]-2-{1-[2-(6-chloro-1,2,3,4-tetrahydroacridin-9-ylamino)ethyl]-1H-1,2,3-triazol-4-yl}acetamide* (**14**). It was prepared as described for **9**. From azide **8** (110 mg, 365 µmol), the triazole derivative **14** (138 mg, 60% yield) was obtained as a brown oil; *R*_f_ (cyclohexane/AcOEt 6:4) 0.10; ^1^H-NMR (300 MHz, DMSO-*d*_6_) *δ* 0.08 [s, 6H, OSi(C*H*_3_)_2_C(CH_3_)_3_], 0.93 [s, 9H, OSi(CH_3_)_2_C(C*H*_3_)_3_], 1.78 (complex signal, 4H, tacrine 2-H_2_, 3-H_2_), 2.59 (t, *J* = 5.8 Hz, 2H, tacrine 1-H_2_), 2.88 (br s, 2H, 2-H_2_), 3.50 (m, 2H, tacrine 4-H_2_), 3.69 (s, 3H, Ph-3-OCH_3_), 3.84 (m, 2H, NH-C*H*_2_-CH_2_-N), 4.19 (s, 2H, CONHC*H*_2_), 4.54 (m, 2H, NH-CH_2_-C*H*_2_-N), 5.75 (N*H*-CH_2_-CH_2_-N), 6.66 (complex signal, 2H, Ph-5-H, Ph-6-H), 6.85 (s, 1H, Ph-2-H), 7.32 (d, *J* = 9.0 Hz, 1H, tacrine 7-H), 7.76–8.03 (complex signal, 3H, triazole 5-H, tacrine 5-H and 8-H), 8.49 (CON*H*CH_2_); ^13^C-NMR (75 MHz, DMSO-*d*_6_) *δ* −4.8 [2CH_3_, OSi(*C*H_3_)_2_C(CH_3_)_3_], 18.1 [C, OSi(CH_3_)_2_*C*(CH_3_)_3_], 22.0 (CH_2_, tacrine C2), 22.3 (CH_2_, tacrine C3), 24.7 (CH_2_, tacrine C4), 25.5 [3CH_3_, OSi(CH_3_)_2_C(*C*H_3_)_3_], 32.7 (CH_2_, C2), 33.2 (CH_2_, tacrine C1), 40.2 (CH_2_, NH-*C*H_2_-CH_2_-N), 47.6 (CH_2_, NH-CH_2_-*C*H_2_-N), 49.6 (CH_2_, CONH*C*H_2_), 55.2 (CH_3_, Ph-3-OCH_3_), 111.5 (CH, Ph-C2), 117.1 (C, tacrine C9a), 118.7 (C, triazole C4), 119.3 (C, tacrine C8a), 119.4 (CH, Ph-C5), 120.1 (CH, Ph-C6), 123.8 (2CH, tacrine C7, triazole C5), 125.2 (CH, tacrine C8), 126.3 (CH, tacrine C5), 132.7 (C, Ph-C1), 132.9 (C, tacrine C-6), 147.1 (C, Ph-C4), 149.9 (C, tacrine C10a), 150.3 (C, Ph-C3), 152.4 (C, tacrine C9), 159.3 (C, tacrine C4a), 168.8 (C, C1); MS (ESI+) 635 [M]^+^.

*N-[4-(hydroxy)-3-methoxybenzyl]-2-{1-[2-(1,2,3,4-tetrahydroacridin-9-ylamino)ethyl]-1H-1,2,3-triazol-4-yl}acetamide* (**15**). Camphorsulfonic acid (34 mg, 146 µmol) was added to a solution of the TBDMS-protected triazole derivative **13** (89 mg, 148 µmol) in MeOH (5 mL), and the reaction mixture was stirred at room temperature for 18 h. The solvent was evaporated at reduced pressure, and the crude product was directly purified by column chromatography (silica gel, AcOEt/MeOH mixtures, gradient elution, from 100:0 to 90:10, 80:20 and 50:50), to afford impure triazole derivative **15**. After re-purification by RP-HPLC (Kromasil, MeCN/H_2_O (0.1% TFA), 40 min, gradient method C), compound **15** (11 mg, 15% yield) was obtained as a light brown solid; *R*_f_ (AcOEt/MeOH 1:1) 0.63; ^1^H-NMR (300 MHz, CD_3_OD) *δ* 1.92–2.05 (complex signal, 4H, tacrine 2-H_2_, 3-H_2_), 2.62 (m, 2H, tacrine 1-H_2_), 3.01 (m, 2H, tacrine 4-H_2_), 3.56 (s, 2H, 2-H_2_), 3.84 (s, 3H, Ph-3-OCH_3_), 4.28 (s, 2H, CONHC*H*_2_), 4.45 (t, *J* = 5.7 Hz, 2H, NH-C*H*_2_-CH_2_-N), 4.77 (t, *J* = 5.7 Hz, 2H, NH-CH_2_-C*H*_2_-N), 6.72 (complex signal, 2H, Ph-5-H, Ph-6-H), 6.85 (s, 1H, Ph-2-H), 7.58 (m, 1H, tacrine 7-H), 7.76–7.89 (complex signal, 3H, triazole 5-H, tacrine 5-H and 6-H), 8.27 (d, *J* = 9.0 Hz, 1H, tacrine 8-H); ^13^C-NMR (75 MHz, DMSO-*d*_6_) *δ* 21.7 (CH_2_, tacrine C2), 22.8 (CH_2_, tacrine C3), 24.9 (CH_2_, tacrine C4), 29.4 (CH_2_, tacrine C1), 33.0 (CH_2_, C2), 44.2 (CH_2_, CONH*C*H_2_), 48.5 (CH_2_, NH-*C*H_2_-CH_2_-N), 51.1 (CH_2_, NH-CH_2_-*C*H_2_-N), 56.4 (CH_3_, Ph-3-OCH_3_), 112.4 (CH, Ph-C2), 114.4 (C, tacrine C9a), 116.1 (CH, Ph-C5), 117.6 (C, tacrine C8a), 120.2 (CH, tacrine C8), 121.3 (CH, Ph-C6), 125.5 (CH, triazole C5), 125.9 (CH, tacrine C7), 126.9 (2CH, tacrine C5 and C6), 131.1 (C, triazole C4), 134.2 (C, Ph-C1), 139.4 (C, Ph-C4), 146.9 (C, Ph-C3), 149.0 (C, tacrine 10a), 152.7 (C, tacrine C9), 158.8 (C, tacrine C4a), 180.1 (C, C1); MS (ESI+) 487 [M]^+^; HRMS (ESI+), calculated for C_27_H_31_N_6_O_3_ [M + H]^+^ 487.2458, found 487.2455; HPLC purity 98.8% (*t*_R_ = 20.6 min, method B).

*2-{1-[2-(6-Chloro-1,2,3,4-tetrahydroacridin-9-ylamino)ethyl]-1H-1,2,3-triazol-4-yl}-N-[4-(hydroxy)-3-methoxybenzyl]acetamide* (**16**). A solution of the TBDMS-protected triazole derivative **14** (114 mg, 179 µmol) in anhydrous THF (1.8 mL) was cooled to 0 °C, and then treated dropwise with TBAF (0.10 mL, 120 µmol). The reaction mixture was stirred at 0 °C for 1 h and at room temperature for an additional 1 h. To the resulting mixture AcOEt (15 mL) and 10% aqueous Na_2_CO_3_ were added. The phases were separated, and the aqueous phase was extracted with AcOEt/THF. The combined organic extracts were dried over anhydrous Na_2_SO_4_, and concentrated at reduced pressure. Purification of the crude product by column chromatography (silica gel, EtOAc/MeOH mixtures, gradient elution, from 100:0 to 90.10, 80:20, and 50:50) afforded the triazole derivative **16** (64 mg, 69% yield) as a green-brown oil; *R*_f_ (AcOEt/MeOH 1:1) 0.63; ^1^H-NMR (300 MHz, CD_3_OD) *δ* 1.82–1.90 (complex signal, 4H, tacrine 2-H_2_, 3-H_2_), 2.55 (m, 2H, tacrine 1-H_2_), 2.89 (m, 2H, tacrine 4-H_2_), 3.58 (s, 2H, 2-H_2_), 3.77 (s, 3H, Ph-3-OCH_3_), 4.06 (t, *J* = 5.4 Hz, 2H, NH-C*H*_2_-CH_2_-N), 4.24 (s, 2H, CONHC*H*_2_), 4.59 (t, *J* = 5.4 Hz, 2H, NH-CH_2_-C*H*_2_-N), 6.68 (complex signal, 2H, Ph-5-H, Ph-6-H), 6.81 (s, 1H, Ph-2-H), 7.29 (dd, *J* = 9.0 Hz, *J′* = 1.5 Hz, 1H, tacrine 7-H), 7.68 (br s, 1H, tacrine 5-H), 7.74 (s, 1H, triazole 5-H), 7.85 (d, *J* = 9.0 Hz, 1H, tacrine 8-H); ^13^C-NMR (75 MHz, DMSO-*d*_6_) *δ* 22.9 (CH_2_, tacrine C2), 23.5 (CH_2_, tacrine C3), 24.8 (CH_2_, tacrine C4), 25.7 (CH_2_, tacrine C1), 33.0 (CH_2_, C2), 33.6 (CH_2_, CONH*C*H_2_), 44.2 (CH_2_, NH-*C*H_2_-CH_2_-N), 51.6 (CH_2_, NH-CH_2_-*C*H_2_-N), 56.4 (CH_3_, Ph-3-OCH_3_), 112.4 (CH, Ph-C2), 116.1 (CH, Ph-C5), 117.5 (C, tacrine C8a), 118.8 (C, tacrine C9a), 121.3 (CH, Ph-C6), 124.8 (2CH, triazole C5, tacrine C7), 126.0 (CH, tacrine C8), 126.5 (CH, tacrine C5), 131.1 (C, triazole C4), 133.5 (C, Ph-C1), 136.6 (C, tacrine C6), 146.1 (C, Ph-C4), 146.8 (C, Ph-C3), 148.9 (C, tacrine 10a), 153.6 (C, tacrine C9), 158.8 (C, tacrine C4a), 171.4 (C, C1); MS (ESI+) 521 [M]^+^; HRMS (ESI+), calculated for C_27_H_30_^35^ClN_6_O_3_ [M + H]^+^ 521.2068, found 521.2072; HPLC purity 95.9% (*t*_R_ = 20.6 min, method B).

### 3.2. Biological Profiling

#### 3.2.1. Evaluation of hAChE and hBChE Inhibitory Activity

The inhibitory activity of the novel compounds on human recombinant AChE and on human serum BChE (Sigma, Milan, Italy) was evaluated spectrophotometrically by the method of Ellman et al. [34]. The AChE stock solution was prepared by dissolving human recombinant AChE lyophilized powder in 0.1% Triton X-100/0.1 M potassium phosphate, pH 8.0. The stock solution of human serum BChE was prepared by dissolving the lyophilized powder in aqueous 0.1% gelatine. The stock solutions of the novel compounds (1 mM) were prepared in MeOH. The assay solution contained 340 μM 5,5′-dithiobis(2-nitrobenzoic acid) (DTNB), 0.02 unit/mL hAChE or hBChE, and 550 μM substrate (acetylthiocholine iodide or butyrylthiocholine iodide, for AChE and BChE, respectively), in 0.1 M potassium phosphate, pH 8.0. Assay solutions with and without inhibitor were preincubated at 37 °C for 20 min, and then the substrate was added. Blank solutions containing all components except the enzymes were prepared in parallel to correct for non-enzymatic hydrolysis of the substrate. Initial rate assays were performed at 37 °C with a Jasco V-530 double beam spectrophotometer. At least five increasing concentrations of the inhibitors, which produced 20–80% inhibition of the enzymatic activity, were assayed. IC_50_ values were calculated using Microcal Origin 3.5 software (Microcal Software, Inc., Darmstadt, Germany).

#### 3.2.2. PAMPA-BBB Assay

The brain permeability (*Pe*) of the target compounds was determined in vitro using the parallel artificial membrane permeation assay for blood-brain barrier penetration described by Di et al. [41], employing a lipid extract of porcine brain membrane in a mixture of PBS/EtOH 70:30. Assay validation was implemented by comparing the experimental and reported *Pe* values of a set of fourteen commercial drugs (Table 2). A good correlation was obtained: *Pe* (exp) = 1.5087 *Pe* (lit) − 0.8974 (*R*^2^ = 0.9296). From this equation, and taking into account the limits established by Di et al. for BBB permeation, the following ranges of permeability were established: high BBB permeation (CNS+): *Pe* (10^−6^ cm s^−1^) > 5.13; low BBB permeation (CNS−): *Pe* (10^−6^ cm s^−1^) < 2.12, and uncertain BBB permeation (CNS±): 5.13 > *Pe* (10^−6^ cm s^−1^) > 2.12.

### 3.3. Structural Biology

#### 3.3.1. Crystallization and Data Collection

*Tc*AChE (12 mg mL^−1^), purified according to Sussman et al. [48], was crystallized by the hanging-drop vapor diffusion method. Equal volumes (1 µL) of protein solution and of 30% PEG 200/50 mM MES, pH 6.0, were mixed at 4 °C. Crystals appeared within a few days, and were harvested after 2–3 weeks. The crystals were then soaked in the above-mentioned mother liquor complemented with the candidate compounds at a concentration of 1 mM, for at least 12 h. All data were collected on the ID30A1 beamline of the European Synchrotron Radiation Facility (ESRF, Grenoble, France) from crystals flash-frozen and stored in liquid nitrogen until utilized.

#### 3.3.2. Data Processing and Refinement

Diffraction images were indexed and integrated using XDS, and intensities were further scaled and merged with XSCALE. Phases were retrieved using the molecular replacement technique with PHASER. PDB entry 2Xi4 served as the starting model of *Tc*AChE that was employed. All data were obtained from orthorhombic *Tc*AChE crystals, and two subunits were placed in the asymmetric unit of all complex structures. The model was refined by iterative cycles of refinement with phenix.refine and model building using Coot. With phenix.refine, refinement of atomic positions and individual isotropic temperature factors was performed in real space (against the experimental electron density map) and in reciprocal space (against experimental intenstities). All ligand topologies were generated with the PRODRG server and their occupancies were refined during the final cycles of refinement. The coordinates and structure factors have been deposited in the Protein Data Bank under accession code 6FOT, 6FOU, and 6FOV, for structures of complexes of **5**, **6** and **16**, respectively, with *Tc*AChE. Data collection and refinement statistics are presented in Table 3.

## 4. Conclusions

Starting from the chemical structures of the potent brain-permeable AChE inhibitors tacrine, 6-chlorotacrine, and huprine Y, which are of interest in the context of Alzheimer’s disease treatment, we have designed and synthesized two series of derivatives of increased polarity with the aim of precluding their penetration into the central nervous system, thus confining their anticholinesterase action to the peripheral level for potential therapeutic use against myasthenia gravis. In one of the series, we increased polarity by the classic approach based on quaternization by alkylation of a nucleophilic nitrogen atom, which produced the quinolinium derivatives of tacrine, 6-chlorotacrine, and huprine Y **4**–**6**. In a second series, we increased polarity by installation of a 1,2,3-triazole ring within a side chain at position 9 of tacrine and 6-chlorotacrine (compounds **9** and **10**), and of an additional polyphenol-like moiety in compounds **15** and **16**. In general, these structural changes lead to decreased AChE and BChE inhibitory activities relative to the parent compounds, with the exception of the triazole-containing compound **16**, which is a more potent AChE and BChE inhibitor than the parent 6-chlorotacrine, likely as a result of multi-site binding within the active-site gorge of the enzyme, as confirmed by the crystal structure of its complex with *Tc*AChE. Despite their lower anticholinesterase potencies compared with the parent compounds, most of the novel derivatives are clearly more potent than pyridostigmine, the preferred drug for symptomatic treatment of myasthenia gravis, and have been found to display low brain permeability values in the PAMPA-BBB assay, thus possessing favorable pharmacodynamic and pharmacokinetic attributes for the intended use against myasthenia gravis.

## Figures and Tables

**Table 1:** hAChE and hBChE inhibitory activities and PAMPA-BBB permeabilities of the target and reference compounds.

Compounds	hAChE IC_50_ (μM) ^1^	hBChE IC_50_ (μM) ^1^	PAMPA-BBB *Pe* (10^−6^ cm/s) (Prediction) ^2^
**4**	12.0 ± 1.00	2.85 ± 0.19	1.70 ± 1.50 (CNS−)
**5**	2.45 ± 0.28	22.3 ± 1.50	1.20 ± 0.80 (CNS−)
**6**	0.06 ± 0.01 ^3^	3.25 ± 0.15	10.90 ± 1.50 (CNS+)
**9**	1.73 ± 0.12	0.17 ± 0.01	0.03 ± 0.001 (CNS−)
**10**	0.20 ± 0.01	3.25 ± 0.17	0.02 ± 0.001 (CNS−)
**15**	0.25 ± 0.01	0.05 ± 0.00 ^4^	0.03 ± 0.001 (CNS−)
**16**	0.01 ± 0.00 ^5^	0.29 ± 0.01	2.97 ± 0.61 (CNS±)
pyridostigmine	1.38 ± 0.07	2.13 ± 0.20	-
tacrine	0.32 ± 0.01	0.05 ± 0.00 ^6^	-
6-chlorotacrine	0.01 ± 0.00 ^7^	0.50 ± 0.03	19.8 ± 0.4 (CNS+)
huprine Y	^8^	0.18 ± 0.01	23.8 ± 2.7 (CNS+)

^1^ IC_50_ (half maximal inhibitory concentration) towards human recombinant AChE and human serum BChE. Values are expressed as mean ± standard error of the mean (SEM) of at least three experiments, each performed in duplicate; ^2^ Permeability values (*Pe*) from the PAMPA-BBB assay and prediction of BBB permeation. Data are the mean of three independent experiments ± SD. High BBB permeation (CNS+) is expected for compounds with *Pe* > 5.19, whereas low BBB permeation (CNS−) is expected for compounds with *Pe* < 2.07; ^3^ IC_50_ hAChE = 59.2 nM (±5.7); ^4^ IC_50_ hBChE = 54.8 nM (±1.6); ^5^ IC_50_ hAChE = 7.18 nM (±0.29); ^6^ IC_50_ hBChE = 45.8 nM (±3.0); ^7^ IC_50_ hAChE = 14.5 nM (±0.9); ^8^ IC_50_ hAChE = 1.07 nM (±0.05).

**Table 2:** Experimental and reported BBB permeability (*Pe* 10^−6^ cm s^−1^) values of the commercial drugs used for assay validation.

Compound	Reported *Pe* ^1^	Experimental *Pe* ^2^
Cimetidine	0.0	0.7 ± 0.1
Lomefloxacin	1.1	0.8 ± 0.1
Norfloxazin	0.1	0.9 ± 0.1
Ofloxazin	0.8	1.0 ± 0.1
Hydrocortisone	1.9	1.4 ± 0.1
Piroxicam	2.5	1.8 ± 0.1
Clonidine	5.3	6.5 ± 0.1
Corticosterone	5.1	6.7 ± 0.1
Imipramine	13.0	12.3 ± 0.1
Promazine	8.8	13.8 ± 0.3
Progesterone	9.3	16.8 ± 0.3
Desipramine	12.0	17.8 ± 0.1
Testosterone	17.0	24.7 ± 1.4
Verapamil	16.0	25.4 ± 1.6

^1^ Taken from [41]; ^2^ Values are expressed as the mean ± SD of three independent experiments.

**Table 3:** Data collection and refinement statistics. ^1^

	5	6	16
Resolution range (Å)	44.04–1.79 (1.854–1.79)	46.03–1.82 (1.82–1.89)	45.7–1.9(1.968–1.9)
Space group	P 21 21 21	P 21 21 21	P 21 21 21
Unit cell (Å)	91.44 106.61 150.53	91.74 106.44 150.68	91.82 106.81 151.67
Total reflections	634,220 (58,489)	642,785 (63,116)	1,072,187 (87,007)
Unique reflections	138,453 (13,737)	130,469 (12,879)	117,742 (11,618)
Multiplicity	4.6 (4.3)	4.9 (4.9)	7.7 (7.5)
Completeness (%)	99.66 (99.75)	98.4 (91.8)	99.71 (99.38)
Mean I/sigma(I)	12.9 (1.5)	12.6 (1.6)	8.8 (1.0)
Wilson B-factor (Å^2^)	24.63	24.09	29.21
R-merge	0.067 (0.943)	0.075 (1.054)	0.128 (1.286)
CC1/2	0.998 (0.686)	0.999 (0.719)	0.998 (0.588)
Reflections used in refinement	138,303 (13,721)	159,670 (14,805)	117,502 (11,589)
Reflections used for R-free	6854 (681)	7990 (773)	5876 (580)
R-work	0.1797 (0.3422)	0.1896 (0.3100)	0.1880 (0.3357)
R-free	0.2125 (0.3788)	0.2193 (0.3114)	0.2177 (0.3411)
Number of non-hydrogen atoms	9962	9719	9640
macromolecules	8591	8583	8564
ligands	321	153	163
solvent	1040	983	916
Protein residues	1067	1064	1068
RMS (bonds) (Å)	0.006	0.007	0.007
RMS (angles) (°)	0.88	0.86	0.88
Ramachandran favored (%)	96.29	96.30	96.03
Ramachandran allowed (%)	3.71	3.70	3.88
Ramachandran outliers (%)	0.00	0.00	0.09
Rotamer outliers (%)	0.74	1.40	2.40
Clashscore	4.19	3.67	3.45
Average B-factor (Å^2^)	31.03	31.94	35.77
macromolecules	29.49	30.88	34.72
ligands	45.26	45.93	50.66
solvent	39.32	38.98	42.98

^1^ Statistics for the highest-resolution shell are shown in parentheses.
